# Applications of Natural Antimicrobials in Food Packaging and Preservation

**DOI:** 10.3390/foods10030568

**Published:** 2021-03-09

**Authors:** Dolores Rodrigo, Alfredo Palop

**Affiliations:** 1 Instituto de Agroquímica y Tecnología de Alimentos (IATA-CSIC), Avd Agustín Escardino Benlloch, 7, Paterna, 46980 Valencia, Spain; lolesra@iata.csic.es; 2 Conservación y Seguridad de Alimentos, Unidad Asociada al CSIC, 46980 Valencia, Spain; 3 Departamento de Ingeniería Agronómica, Instituto de Biotecnología Vegetal, Campus de Excelencia Internacional Regional “Campus Mare Nostrum”, Escuela Técnica Superior de Ingeniería Agronómica, Universidad Politécnica de Cartagena, 30202 Cartagena, Spain

In the food science field, the term “antimicrobial” basically refers to active substances of synthetic or natural origin, that are directly or indirectly present in a specific food, packaging material or food contact surface that affect the viability or the growth of microorganisms in that matrix. Preserving food by applying synthetic preservatives such as weak organic acids, oxidizing or polymeric substances has been widely used in the food industry. However, in the last years, increasing concerns over negative effects on health due to those synthetic preservatives, have motivated the scientific community to focus its efforts on natural preservatives. Nevertheless, it is still necessary to carry out in-depth studies to be able to unravel numerous effects and potentialities hitherto unknown. Consequently, this special issue deals with research manuscripts on the use of natural antimicrobials for food preservation and food packaging.

This editorial aims to concisely discuss and summarize the results collected in the current Special Issue based on various aspects considered relevant such as the origin of the antimicrobial (e.g., plant, microbial, algae), the type of antimicrobial test that has been carried out, and the antimicrobial application (food ingredient or as packaging material).

Natural antimicrobials have been identified and isolated from plants, animal and bacterial sources. Plant-based antimicrobials are often found in the essential oils of plants. These oils are obtained from flowers, seeds, fruits, buds, leaves, branches, bark, wood, bulbs, rhizomes or roots and are defined as aromatic mixtures of secondary metabolites most of them involved in the defense of plants against phytopathogens, herbivores and insects. In this special issue, Somrani et al. [1] studied the antibacterial and anti-biofilm activity of cinnamon (EOC), onion (EOO) and garlic (EOG) essential oils. The major components identified by GC-MS for EOG and EOO were organo-sulfur compounds regarded as thiosulfates, specifically diallyl trisulfide (25.13%) and diallyl disulfide (22.74%) and trisulfide dipropyl (35.46%) and dipropyl disulfide (31.11%) for EOG and EOO, respectively. EOC was mainly composed of E-cinnamaldehyde (76.54%), which belongs to the family of phenylpropanoids. Djenane et al. [2] studied the impact of a combined treatment with *Olea europaea* subsp. *laparrinei* leaf extracts (*laper*.OLE) with nisin to improve shelf-life of camel steaks. Total polyphenol content of *laper*.OLE samples was 216.5 ± 2.9 mg GAE/100 g. Further HPLC-DAD analysis revealed the presence of seven main compounds of which oleuropein is the main component (63.03%), followed by luteolin-7–glucoside (11.28%), apigenin-7-glucoside (8.15%), and hydroxytyrosol (5.93%). Motelica et al. [3] obtained biodegradable antimicrobial films to be used as packaging material. Those films were based on chitosan, as polymer, with ZnO and Ag nanoparticles as filler/antimicrobial agents that were loaded with citronella essential oil in order to increase the antimicrobial activity of the films. Main components of citronella were geraniol, citronellal, and citronellol as its major constituents. Amor et al. [4] developed polymeric chitosan films with basil essential oil microencapsulated for packaging material. The GC-FID analysis of basil EO reported that the component most present by far was linalool (41.3%) followed by 1,8-cineole (9.6%), (Z)-isoeugenol (5.9%), 1-epi-cubenol (4.8%), α-transbergamotene (4.6%), and (Z)-anethol (3.2%). Jaśkiewicz et al. [5] obtained and evaluated the properties of biodegradable starch film with the addition of phytic acid (0.05%) as a cross-linking agent and chicory root extract (1–5%) as an antimicrobial agent. Chicory was extracted by different methods obtaining water, water/methanol, and water/enzyme extracts that were analyzed (UHPLC-ESI-MS) to determine the main groups of compounds showing antimicrobial properties. Sesquiterpene lactones and polyphenols were found to be the most active groups for antimicrobial activity, being the water-enzyme extract the one that showed higher sesquiterpene lactones and polyphenol content.

The special issue also includes studies of natural antimicrobials from bacterial sources such as nisin [2]. Nisin is a normal food constituent in meat and fermented dairy products due to the presence of many lactic acid bacteria, including *Lactococcus lactis* subsp. *lactis*. It is well-documented for its inhibitory effect against various microorganisms. Algae are an alternative source of bioactive compounds among which are antimicrobials. Martelli et al. [6] obtained antimicrobial extracts from four species of seaweed (*Himanthalia elongata, Laminaria* spp., *Palmaria palmata* and *Undaria pinnatifida*) and a cyanobacterium (*Arthrospira platensis*). Total phenolic content (TPC) of samples was determined by a standard spectrophotometric method (Folin-Ciocalteu). Among the samples tested, *U. pinnatifida* presented the lowest phenolic content (0.14 ± 0.01 mg GAE/g), while the highest was found in *H. elongata* extracts (18.79 ± 1.90 mg GAE/g). Extracts obtained from *P. palmata* and the cyanobacterium *A. platensis* showed comparable phenolic content, with values of 2.45 ± 0.06 mg GAE/g and 3.18 ± 0.48 mg GAE/g, respectively. Those values are much lower than the ones reported for *laper*.OLE [2] but could be related with the antimicrobial activity of algae samples [6].

Another valuable source for natural antimicrobials are by-products of the food processing industry. As a result of the agri-food industry activity, large amounts of waste with no, a priori, value are produced. However, its elimination implies a highly negative impact, both economic and environmental. That is why the revaluation of those wastes into by-products is one of the main objectives of the European Union to increase food chain sustainability. Lee et al. [7] studied the fabrication conditions of functional (antioxidant and antibacterial) bioelastomers using by-products from the aronia (*Aronia melanocarpa*) juice processing. Antioxidant activity of aronia extracts was characterized by determining TPC, total flavonoid content, DPPH Radical Scavenging Activity and ABTS Radical Scavenging Activity of the extracts. 37.2 ± 1.3 mg GAE/g dry powder polyphenols, and 18.5 ± 0.8 mg RE/g dry powder flavonoids indicated that aronia processing by-products are an effective resource for antioxidant materials. As for scavenging activity, aronia by-product showed 63.8 ± 0.1% and 29.4 ± 0.3% radical scavenging activity by DPPH and ABTS, respectively. Authors related this remarkable antioxidant activity with the antimicrobial activity of aronia bioelastomers. 

These natural compounds may have very different antimicrobial applications, as shown in the variety of uses proposed in the manuscripts included in this special issue. Somrani et al. [1] applied them to fight biofilms formed in the food processing industries. Djenane et al. [2], Amor et al. [4] and Martelli et al. [6] added the antimicrobials directly to the foods, while Motelica et al. [3], Jaśkiewicz et al. [5] and Lee et al. [7] included them in the packaging material.

There are very different methods to test the antimicrobial efficiency of these compounds. For example, Somrani et al. [1] used the agar diffusion and the macrobroth dilution assays to test the effect of essential oils on *Listeria monocytogenes* planktonic cells and they used the microculture method, in microtiter plates, to check their antibiofilm effects in the same microorganism, for both inhibiting initial cell attachment and biofilm eradication. Amor et al. [4] also used the agar diffusion assay to test the antimicrobial properties of their basil essential oil against different bacteria (*Brochothrix thermosphacta, Carnobacterium maltaromaticum, Enterococcus faecalis, Staphylococcus xylosus, Staphylococcus saprophyticus, Listeria innocua, Streptococcus salivarius, Hafnia alvei, Serratia proteamaculans* and *E. coli)* and the macrobroth dilution only against the microorganisms that showed a strong sensitivity in the agar diffusion test. Amor et al. [4] also tested the minimum lethal concentration of basil essential oil by plating the cultures without visible bacterial growth onto Tryptone Soy Agar. Agar diffusion was also used by Martelli et al. [6] to test the in vitro efficacy of the antimicrobials extracted from their algae species against several foodborne pathogens (*Salmonella* spp., *L. monocytogenes, Escherichia coli, Staphylococcus aureus* and *Bacillus cereus*). On their side, Jaśkiewicz et al. [5] used the microculture method, to check the antibacterial effect of chicory root extracts against *Pseudomonas fluorescens, E. coli, S. aureus* and *Bacillus subtilis.*

When the antimicrobials are incorporated into the packaging material, agar diffusion assay was also used by most of the authors. Lee et al. [7] checked the antibacterial properties of their bioelastomer fabricated from aronia juice processing by-products against *S. aureus* and *E. coli*, by just placing pieces of the bioelastomer onto the agar plates. Jaśkiewicz et al. [5] also used this method to test the biodegradable starch films they developed incorporating chicory root extracts as antimicrobial against *P. fluorescens, E. coli, S. aureus, B. subtilis* and two fungi, *Candida albicans* and *Aspergillus niger.* Motelica et al. [3] checked the antimicrobial activity of their biodegradable chitosan films with ZnO and Ag nanoparticles loaded with citronella essential oil against *C. albicans, S. aureus and E. coli* by means of placing film strips onto the agar plates and measuring inhibition zones after incubation. Amor et al. [4] tested their edible chitosan based films grafted with microencapsulated basil essential oil on *S. saprophyticus* and *E. coli* using a different assay, which consisted of incubating a piece of the film together with the bacterial growing culture in the same test tube, allowing for the interaction of the film and the bacteria and evaluating the viable count after incubation by plating.

When the antimicrobials or the antimicrobial packaging films are applied directly to foods, other methods may be more appropriate to estimate the antimicrobial properties. Djenane et al. [2] observed the changes in several physical, chemical and microbiological properties of camel meat to check for the efficacy of the mixture of *laper*.OLE with nisin (pH, lipid oxidation through the thiobarbituric acid method, pigment oxidation measuring the percentage of metmyoglobin, cutting action force using a Warner-Bratzler shear device and total psychrotrophic and *Pseudomonas* spp. counts), and recalled on the sensory analysis carried out with a semi-trained panel to look for the overall acceptance of the meat through storage during 30 days at 1 °C under modified atmosphere packages (80% O_2_ and 20 % CO_2_). Martelli et al. [6] performed microbial challenge tests on salmon tartare inoculated with a cocktail of three strains of *L monocytogenes*. Amor et al. [4] also tested the antimicrobial effect of their film in a real food system, using it to wrap cooked ham, and looking at the evolution of the total aerobic mesophilic bacteria, mesophilic lactic acid bacteria, enterobacteria and yeasts on a 10 days’ storage at 4 °C. The pH of the cooked ham was also measured along incubation. Finally, Motelica et al. [3] only performed a preliminary visual quality check when using their chitosan films with ZnO and Ag nanoparticles loaded with citronella essential oil to package white grapes that were stored at room temperature for 14 days.

The incorporation of the antimicrobials into the packaging films may change their properties, so this is a worth to explore issue. Lee et al. [7] found no changes in the surface and chemical properties of their bioelastomer when aronia juice processing by-products were included, although tensile strength was not appropriate when the mixture contained 50% aronia powder. An increase in the thickness, tensile strength and opacity [4,5] and a decrease in water vapor permeability [3,5] has been observed when adding antimicrobials into the packaging films.

All the compounds tested have shown antimicrobial properties. Somrani et al. [1] observed that all essential oils tested were very effective in preventing biofilm formation by *L. monocytogenes*, although effectivity decreased on already preformed biofilms. Djenane et al. [2] found significantly lower microbial counts on camel steaks treated with the combination of *laper*.OLE and nisin than on untreated steaks. Sensory evaluation revealed low initial bitterness defects of the camel steaks treated with the antimicrobials, but this bitterness decreased over storage time. Martelli et al. [6] showed that the effect of algae extract on *L. monocytogenes* in salmon tartare was bacteriostatic, and even bactericidal when the concentration of algae extract was increased. However, Jaśkiewicz et al. [5] only reached to find bacteriostatic effects when chicory root extract was incorporated into their starch film, and the fungistatic effect was even weaker. On the other side, Motelica et al. [3] found enhanced antifungal activity of their chitosan films when ZnO and Ag nanoparticles loaded with citronella essential oil was added, and Amor et al. [4] even found bactericidal effects when the basil essential oil was microencapsulated. Lee et al. [7] found not only antimicrobial, but also antioxidant properties when aronia juice processing by-products were incorporated into their bioelastomer.

The manuscripts compiled in this special issue highlight the potential of numerous natural substances as antimicrobials to be used by the food industry in different applications, and make us reflect on the importance of looking back at the natural environment as a source of bioresources.

## Data Availability

Not applicable.
